# 

**Published:** 1856-10

**Authors:** 


COMMUNICATIONS FOR PUBLICATION TO BE ADDRESSED TO 'EDITORIAL COMMITTEE.'

### IOWA MEDICAL JOURNAL.

CONDUCTED BY THE FACULTY OF THE

COLLEGE OF PHYSICIANS AND SURGEONS

OF THE

IOWA UNIVERSITY.

SMITHSONIAN INSTITUTION. 13596.

PUBLISHED EVERY ALTERNATE MONTH.

TERMS—TWO DOLLARS IN ADVANCE.

VOL. III. SEPTEMBER AND OCTOBER, 1856. NO. 5.

EDITORIAL COMMITTEE,

D. L. M'GUGIN, M. D.

JNO. R. ALLEN, M. D.

FINANCE COMMITTEE.

J. C. HUGHES, M. D.

KEOKUK, IOWA.

PRINTED AT THE POST BOOK AND JOB OFFICE.

BUSINESS COMMUNICATIONS TO THE 'FINANCE COMMITTEE.'

